# Hijacking the Host Cell for Replication: Pro-Viral Host Factors Involved in EVA71 Infection

**DOI:** 10.3390/ijms26167992

**Published:** 2025-08-19

**Authors:** Qian Wang, Xing Wu, Mingchen Liu, Lu Li, Ying Wang, Qian He, Xuanxuan Zhang, Zhenglun Liang, Fan Gao, Xiao Ma

**Affiliations:** 1 State Key Laboratory of Drug Regulatory Science, NHC Key Laboratory of Research on Quality and Standardization of Biotech Products, NMPA Key Laboratory for Quality Research and Evaluation of Biological Products, Research Units of Innovative Vaccine Quality Evaluation and Standardization, Chinese Academy of Medical Sciences, National Institutes for Food and Drug Control, Beijing 102629, China; qwang187@126.com (Q.W.); eastarwx@163.com (X.W.); liumingchen79@126.com (M.L.); lilulilu@nifdc.org.cn (L.L.); wangying7586@nifdc.org.cn (Y.W.); hq5740@126.com (Q.H.); zhangxx_01@163.com (X.Z.); lzhenglun@126.com (Z.L.); 2 School of Life Sciences, Tianjin University, Tianjin 300072, China

**Keywords:** Enterovirus A71, hand foot and mouth disease, viral replication, pro-viral factors, antiviral drugs, vaccines

## Abstract

Enterovirus A71 (EVA71) is a major pathogen that causes hand, foot, and mouth disease (HFMD). Although the symptoms of HFMD can be self-limiting, severe meningitis, encephalitis, myocarditis, and acute flaccid paralysis may occur. Upon EVA71 infection, the host cells deploy an intricate network of factors to orchestrate cellular responses and maintain cellular homeostasis. However, the virus has evolved various strategies to avoid unfavorable host restrictions and to establish a productive infection process. As response regimens are correlated with disease symptoms, exploring the interactions between the virus and host contributes to understanding the pathogenesis and underlying mechanisms of infection. In this review, we summarized the recent research progress related to pro-viral factors during EVA71 infection and discussed the underlying mechanisms employed by EVA71 to facilitate virion production. These insights may help identify antiviral therapeutic candidates and support vaccine development.

## 1. Introduction

Hand, foot, and mouth disease (HFMD) is a globally prevalent infectious disease, but it is mainly reported in the Asia-Pacific region, where epidemics reoccur every 2–3 years [1,2,3]. Enterovirus (EV) A71 (EVA71) is a major etiological microorganism of severe HFMD and fatal neurological diseases in infants, young children, and immunocompromised individuals [2,3]. EVA71 infection can also induce herpes angina, meningitis, encephalitis, myocarditis, respiratory diseases, and acute flaccid paralysis [4]; thus, it poses a significant threat to global public health.

**Figure 1:**
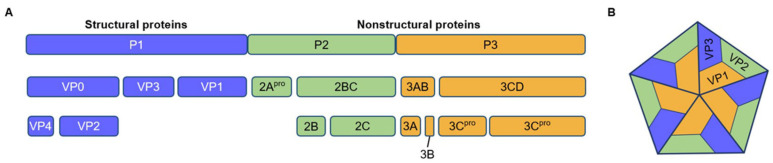
Structure of the EVA71 virion: (**A**) The large viral polyprotein undergoes primary cleavage by viral proteases 2A^pro^ and 3C^pro^ into three precursors: P1 (structural proteins), P2 (non-structural proteins), and P3 (non-structural proteins). P1 is further processed by 3CD or 3C^pro^ to generate VP0, VP3, and VP1. VP0 undergoes autocatalytic cleavage to produce VP2 and VP4 during virion maturation. Concurrently, 3C^pro^ cleaves P2 into 2A^pro^ and 2BC (later processed to 2B and 2C), and P3 into 3AB (further cleaved to 3A and 3B/VPg) and 3CD (processed to 3C^pro^ and 3D^pol^). (**B**) The icosahedral particle (20–30 nm diameter) of EVA71 comprises 60 copies each of VP1–VP4. VP1–VP3 form the outer capsid shell. VP4, modified by N-myristoylation, is internally positioned and interacts with the packaged viral RNA and 3B.

EVA71 is a non-enveloped virus that belongs to the *Enterovirus* genus of the *Picornaviridae* family. This virus harbors a 7.4 Kb single-stranded positive-sense RNA (+ssRNA) genome flanked by 5′ and 3′ untranslated regions (UTR). The 5′ UTR contains six stem loops (SLs) [5]. In coordination with canonical initiation factors and internal ribosome entry site (IRES) transfactors (ITAFs), the SLs facilitate genome replication and cap-independent translation via type I IRES [6,7,8]. The single open reading frame encodes a large polyprotein (P1-P3) that can be cleaved into four viral structural proteins (VP1-VP4) and seven non-structural proteins (2A–2C and 3A–3D). VP1-VP3 form a 20–30 nm icosahedral capsid that encapsulates viral RNA, whereas N-myristoylated VP4 is located inside the virion (Figure 1) [9,10]. The virus life cycle comprises attachment, endocytosis, uncoating, genome release, translation, proteolytic processing, genome replication, assembly, and virion maturation and release [4]. Specifically, upon binding to its receptor and subsequent exposure to low endosomal pH, EVA71 undergoes an uncoating process with irreversible conformational changes, resulting in the externalization of VP4 and the N-terminus of VP1, and the viral genome is subsequently released into the cytosol [11,12,13,14]. Mediated by IRES and ITAFs, the viral genome is translated by cellular translation machinery [15]. The polyprotein is proteolytically cleaved into VP1-VP4, 2A-2C, and 3A-3D by the viral proteinases 2A^pro^, 3C^pro^, and 3CD^pro^, and then the viral RNA-dependent RNA polymerase 3D mediates viral replication in a UTR-dependent manner [16,17]. RNA packaging, along with virion maturation, is mediated by heat shock protein (HSP) 90 [18,19]. As a non-enveloped virus, EVA71 can be released from host cells in both a cytolytic and non-lytic manner, with virions wrapped in exosomes facilitating its infection [20].

Notably, there are no effective therapeutic drugs available that target EVA71, as the knowledge gaps in the pathogenesis and underlying molecular mechanisms during EVA71 infection remain largely enigmatic, posing a significant public health threat and economic burden. Elucidating the host–virus interactions involved in infection could reveal novel therapeutic targets and inform vaccine design. In this review, we summarized the recent advances in pro-viral host factors, which are host-derived elements that directly or indirectly facilitate viral processes—including RNA stability/replication, protein translation/stability/enzymatic catalysis, virion assembly/maturation, or creation of proviral cellular environments—thereby enhancing viral replication. The underlying mechanisms of how host determinants contribute to infection are also addressed.

## 2. Pro-Viral Host Factors Facilitating Viral Entry

Receptor-mediated attachment is the initial step in EVA71 infection, involving the post-translational modification of host receptors catalyzed by certain pro-viral factors. A genome-wide CRISPR-Cas9 knockout screen identified several critical host factors, including scavenger receptor class B member 2 (SCARB2), solute carrier family 35 member B2 (SLC35B2), and beta-1, 3-glucuronyltransferase 3 (B3GAT3) as pro-viral factors facilitating EVA71 replication [21]. SLC35B2 or B3GAT3 knockout significantly impairs viral binding and cellular internalization [21]. Mechanistically, SLC35B2, which encodes a transporter of the sulfate donor 3′-phosphoadenosine-5′-phosphosulfate, regulates the tyrosine sulfation of the EVA71 receptor SCARB2 and attachment receptor heparan sulfate (HS) via tyrosylprotein sulfotransferase-dependent sulfate donation [12,21,22]. Concurrently, B3GAT3 is essential for the biosynthesis of the HS backbone, further highlighting the coordinated action of these pro-viral factors in establishing productive EVA71 infection [21].

## 3. Pro-Viral Host Factors Regulating Viral RNA Dynamics

During viral infection, host cells evolve unique subcellular compartmentalization structures, such as replication organelles (ROs) and P-bodies, to cope with disruptions in homeostasis [23,24,25]. Enteroviruses, including EVA71, have evolved sophisticated mechanisms to adopt these structures for viral genome replication. ROs are organelle-like cytoplasmic vesicles enriched with +ssRNAs and viral/host proteins required for replication. Owing to the high concentrations of RNAs and proteins, ROs provide an effective platform for translation and assembly [23,24]. Protected by the bilayer membranes of ROs, viral RNAs, proteins, and intermediates can escape immune surveillance. The viral 3A protein in several enteroviruses recruits phosphatidylinositol-4-kinase IIIβ (PI4KB) to the Golgi apparatus, where it promotes phosphatidylinositol 4-phosphate (PI4P) lipid synthesis and the formation of PI4P-enriched ROs, thereby promoting viral replication [26,27,28]. ADP-ribosylation factor 1 (Arf1) guanosine triphosphatase (GTPase) and its Golgi Brefeldin A-resistant guanine nucleotide exchange factor 1 (GBF1) protein or acyl-coenzyme A-binding domain containing 3 (ACBD3) are required for PI4KB recruitment [28]. In addition, N-terminal acetyltransferase (NAT) 6 enhances viral replication by stabilizing the PI4KB recruiter ACBD3 by blocking its autophagic degradation [29]. Furthermore, the RO-resident protein Annexin A2 (ANXA2) exploits the functions of PI4P induction and 3D recruitment to enhance viral propagation [30].

P-bodies are cytoplasmic ribonucleoprotein (RNP) granules enriched with RNA-binding proteins that ameliorate the surge of RNA induced by abnormal conditions, thereby maintaining cytosolic homeostasis [25,31,32,33]. +ssRNA viruses disrupt the integrity of P bodies and repurpose their components for viral replication [32,33,34]. Viral protease 2A inhibits P-body formation through its proteolytic activity while simultaneously co-opting the P-body constituents DEAD-box helicase (DDX) 6 and eIF4E-transporter (4E-T) to enhance viral RNA synthesis [35]. This strategic repurposing of host RNA-binding proteins represents a novel mechanism for enhancing viral replication.

In addition to subcellular compartmentalized regulation, viral RNA replication is implemented by the RNA polymerase 3D, which can be regulated by altering protein–protein interactions and/or protein-RNA interactions. Interactions between the m6A methyltransferase-like 3 (METTL3) and 3D modulate EVA71 replication [36]. Despite the lack of evidence of RNA methylation activity, restored *mettl3* expression results in increased viral replication compared with that of *mettl3*^−/−^ viruses. Furthermore, METTL3 was found to induce the sumoylation and ubiquitination of 3D, promoting its stability and viral replication [37]. Additionally, chemical modifications play crucial roles in the metabolism and function of EVA71 RNAs. N-acetyltransferase 10 (NAT10)-catalyzed ac4C in the 5′ UTR recruits poly(rC)-binding protein 2 (PCBP2) to stabilize the interaction of viral RNA and 3D [38,39]. This modification-dependent mechanism significantly enhances the efficiency of viral genome replication.

## 4. Pro-Viral Host Factors Modulating Viral Protein

Viruses have evolved several strategies to activate viral translation or inhibit degradation of viral proteins to promote virion accumulation. The DEAD-box family RNA helicase DEAD-box helicase 3 X-linked (DDX3X) unwinds the highly structured secondary structure of domain VI to facilitate ribosome entry [40]. Through the 563 to 565 amino acids, DNA damage-inducible protein 34 (GADD34) interacts with the non-structural protein 3CD and promotes the IRES activity of EVA71 independently of its canonical eukaryotic translation initiation factor 2α (eIF2α) phosphatase activity [41]. Notably, 3CD upregulates both the RNA and protein levels of GADD34, which reinforces this proviral mechanism. The regulation of ITAFs, such as heterogeneous nuclear ribonucleoprotein A1 (hnRNP A1) and polypyrimidine tract-binding protein 1 (PTB), can enhance IRES activity. Cytoplasmic translocation of hnRNP A1 is facilitated by HSP27, which is selectively induced by EVA71 when the expression of the chaperone proteins endoplasmic reticulum (ER) protein 57 and Hsp70 is decreased [42,43]. HSP27 boosts the cytoplasmic translocation of ITAF and promotes the cleavage of 2A^pro^-mediated eukaryotic initiation factor 4G to activate IRES-mediated translation [42]. Similarly, the cytoplasmic translocation of PTB, a newly identified ITAF, is induced during EVA71 infection, which promotes EVA71 viral protein expression and viral propagation [44].

Post-translational modifications also regulate IRES activity. In addition to the ac4C modification, EVA71 RNAs harbor m5C modifications catalyzed by NOP2/Sun RNA methyltransferase family 2 (NSUN2) [45,46]. m5Cs in IRES increase both the stability and translational efficiency of EVA71 RNAs. Moreover, NSUN2 interacts with the VP1 protein, which inhibits VP1 ubiquitination and increases its stability. Additionally, c-FOS, a subunit of the AP-1 transcription factor, can be induced by EVA71 infection via the endogenous IRES by ITAFs, forming a positive feedback loop that boosts EVA71 replication [47,48].

Regulation in autophagy and ubiquitination subvert viral protein degradation inhibition. The ER-resident acetyltransferase NAT8 interacts with EVA71 2B, 3AB, and 3C proteins and modifies lysine acetylation to inhibit the autophagy–lysosome degradation of those viral proteins, which is essential for EVA71 infection [49]. Ubiquitin-mediated degradation is blocked during viral replication. Ubiquitin-specific protease (USP) 21 deubiquitinates K48-linked poly-Ub chains on 2A^pro^, thereby erasing the K48-poly-ub-26S proteasomal degradation signals [50].

Viruses strategically co-opt host proteolytic machinery to mediate the precise cleavage of viral protein precursors, thereby driving the maturation of functional viral proteins. In a previous study, using activity-based probes (ABPs) based on the 3C^pro^ inhibitor Rupintrivir, autophagy-related protein (ATG) 4 was screened and found to have protease activity similar to that of EVA71 3C^pro^ [51]. Notably, functional ATG4B—rather than its proteolytically inactive C74A mutant—restores RNA replication in 3C^pro^-deficient viral mutants, revealing an unprecedented host–virus interplay wherein cellular proteases can functionally compensate for impaired viral protease activity.

Finally, RAS-associated protein 11A (RAB11A) interacts and co-localizes with structural and non-structural viral components, acting as a scaffold that recruits chaperones to facilitate viral maturation and assembly independent of GTPase activity [52].

## 5. Host Metabolic Pro-Viral Factors

Emerging evidence has demonstrated that EVA71 extensively rewires host metabolic pathways, including the tricarboxylic acid (TCA) cycle, glycolysis, and lipid metabolism, to facilitate viral replication [53,54,55]. However, the mechanism by which these metabolites regulate EVA71 replication remains unclear [56,57]. It’s revealed that aspartate, a critical TCA cycle substrate, positively regulates EVA71 replication [58,59,60]. EVA71 infection recruits mTOR to lysosomes to promote ras homolog enriched in brain (RHEB) interaction and activation via the phosphoinositide 3-kinase (PI3K)/protein kinase B (AKT)/Tuberous sclerosis complex 2 axis. This induces the expression of the aspartate transporter protein–solute carrier family 38 member 8 (SLC38A8), leading to enhanced aspartate aminotransferase activity and elevated aspartate expression and uptake [58,59,61]. Depending on SLC38A8, aspartate further triggers the phosphorylation and activation of the mTOR downstream factors ribosomal protein S6 kinase beta-1 (p70S6K1) and ribosomal protein S6 (rpS6), which promote EVA71 replication by facilitating protein translation [61]. Lai et al. reported that EVA71 infection can elevate hyperglycemia in humans and in an hSCARB2-Tg mouse model, potentiating the neurovirulence of EVA71 via hyperglycemia-induced miR-206 expression in the brainstem [62]. miR-206 represses the protein expression of stress granule assembly factor 2 (G3BP2), an RNA-binding protein that attenuates translation, to facilitate IRES-driven viral replication [62,63].

Meanwhile, an altered level of lipid metabolism has been reported to regulate viral replication, as attenuated lipid metabolism inhibits EVA71 replication despite the fact that infection decreases neutral and polar lipids conversely [64]. Oxysterol-binding protein (OSBP), an important mediator the cellular lipid homeostasis and signal transduction, contributes to ROs development and unesterified cholesterol accumulation upon EVA71 infection, which enhances 3AB cleavage and viral plus-strand RNA synthesis [65]. Kobayashi et al. also investigated the crystal structure of OSBP, which underlies the expected function analysis [65].

Additionally, 2B interacts with voltage-dependent anion channel 3 (VDAC3) on the mitochondria periphery to enhance mitochondrial reactive oxygen species (ROS) production, leading to mitochondrial dysfunction and disrupted redox metabolism homeostasis [66]. Although treatment with mitochondrion-specific antioxidant Mito-TEMPO could inhibit EVA71 replication substantially, the precise mechanism by which VDAC3-mediated ROS production promotes EVA71 replication—whether through direct facilitation of viral genome replication/assembly or indirect suppression of host antiviral signaling—requires further investigation.

## 6. Pro-Viral Host Factors Orchestrating Immune Evasion

Immune responses are correlated with disease symptoms [67]. EVA71 has evolved sophisticated mechanisms to evade innate immunity, particularly by disrupting pattern recognition receptor-mediated interferon (IFN) responses through three principal strategies: (i) proteolytic cleavage of critical immune signaling transductors, such as retinoic acid-inducible gene I (RIG-I), mitochondrial antiviral-signaling protein (MAVS), IFN response factor (IRF7), and IFN receptor 1; (ii) impeding protein interactions to affect processes such as translation, activation, or translocation; (iii) upregulation of the expression of host negative-regulators like USP19 and microRNAs to suppress immune responses [68,69,70]. In the following sections, we elaborate on additional immune evasion strategies that were not included in the comprehensive review by Wei et al. [68].

Ras GTPase-activating protein-binding protein (G3BP1) is an RNA-binding protein that regulates RNA stability and translation [71]. Although many viruses directly target G3BP1 [72,73,74], EVA71 not only degrades G3BP1, thereby suppressing its downstream induction of RIG-I and melanoma differentiation-associated protein 5, but also promotes the induction of leucine-rich repeat-containing 25 (LRRC25) expression to degrade both G3BP1 and RIG-I through autophagy [75,76]. Viral 3A recruits LRRC25 and is a pro-viral factor in this process.

The viral structural protein VP1 binds to and triggers autophagic degradation of the m6A reader YTH N6-methyladenosine RNA binding protein F2 (YTHDF2), resulting in elevated levels of GTP binding protein 4 (GTPBP4) [77]. GTPBP4 acts as a negative regulator of type I IFN that inhibits IRF3 binding to the *Ifnb* promoter [77]. EVA71-infection upregulates miR-545 expression to antagonize the functions of phosphatase and tensin homolog (PTEN) and tumor necrosis factor receptor-associated factor (TRAF) 6, thereby relieving the inhibition of IFN signaling [78]. Similarly, miR-628-5p is a pro-viral factor, as it targets TRAF3 to impair the activation of the innate immune system [79].

## 7. Pro-Viral Host Factors Mediating Virion Egress and Dissemination

EVA71 exploits extracellular vehicles (EVs) for viral packaging and dissemination. EVs are conventionally classified into four distinct subtypes based on their diameter: exosomes (50–150 nm), microvesicles (MVs, 100–1000 nm), apoptotic bodies (500–2000 nm), and oncosomes (100–400 nm) [80].

Recent publications have demonstrated that EVA71 is specifically incorporated into exosomes and MVs, forming infectious complexes that exhibit significantly enhanced blood–brain barrier (BBB) penetration compared to free virions [81,82]. Combined with the documented increase in EV concentrations in the sera of patients with severe EVA71 and HFMD, these observations provide compelling evidence for the role of EV-mediated transmission in EVA71 neuroinvasion and pathogenic mechanisms involving the central nervous system (CNS) [83,84]. EVA71-harboring exosomes and MVs are released from host cells when lipid membranes provide effective protection against the host immune system. Mechanistically, exosomes downregulate PI3K/Akt signaling to reduce the expression of the downstream tight junction protein zonula occludens-1 (ZO-1) [81,82]. Simultaneously, MVs reduce ZO-1 and Occludin expression, resulting in compromised BBB integrity [81,82]. These synergistic effects enable efficient viral infection of brain microvascular endothelial cells, whereas concomitant mitochondrial damage exacerbates neuropathology via the nicotinamide adenine dinucleotide phosphate oxidase 4-ROS pathway [81,82]. In addition, 3A can interact with vacuolar protein sorting (VPS) 25, a component of endosomal sorting complex required for transport II that promotes exosome biogenesis and secretion [85], which contributes to rigorous viral replication [83].

miR-4516 is another pro-viral factor, targeting membrane permeability and antagonizing the translation of *poliovirus receptor-related 1 (pvrl1)* mRNA to disrupt cell adhesion molecules at adherens junctions (AJs) [86,87]. However, miR-4516 expression is inhibited during EVA71 infection [86].

The mechanism by which EVA71 exploits the host autophagy machinery to facilitate viral replication was also elucidated recently [88]. Although apoptotic bodies typically originate from programmed cell death pathways, EVA71 has evolved mechanisms to incorporate autophagic processes into its replication cycle [89]. A central regulator of this process is BCL-2 interacting protein 1 (BECLIN1), which is essential to ATG protein recruitment [90,91,92,93,94,95]. Its normal function is frequently associated with viral infections. Specifically, the viral 3D protein interacts directly with BECLIN1, forming a functional complex that enhances viral replication while maintaining steady-state *beclin1* mRNA and protein levels [96]. High mobility group box 1 (HMGB1) is another factor whose expression is induced by infection, and HMGB1-mediated autophagy is dispensable for EVA71 replication in several cell populations [97]. In addition, the further autophagosome-MVB fusion represents a crucial step in the generation of infectious exosomes containing viral RNA, thereby facilitating both viral dissemination and immune evasion [98]. The precise molecular interactions between viral components and the host autophagy machinery are an active area of investigation, with researchers paying particular interest to how EVA71 balances autophagy modulation to promote replication while avoiding premature host cell death.

Current evidence suggests that EVA71-induced apoptosis facilitates viral particle dissemination; however, the molecular mechanisms governing selective viral packaging remain unclear [99]. Viral proteases 2A and 3C serve as the principal inducers of apoptosis through several mechanisms: (i) caspase-mediated cleavage of critical host cell substrates, (ii) disruption of mitochondrial membrane integrity, and (iii) modulation of B-cell lymphoma 2 family protein expression [100,101,102,103,104]. Collectively, these findings suggested that EVA71 has evolved to regulate apoptotic pathways, maintain host cell viability during early infection to support viral replication, and trigger programmed cell death to promote efficient viral release and dissemination. More evidence underlying these mechanisms is warranted for further investigation, as this represents a crucial aspect of EVA71 pathogenesis with potential therapeutic implications.

## 8. Other Pro-Viral Factors

Certain autophagy-associated factors can modulate EVA71 replication via mechanisms independent of canonical autophagic functions. For example, heat shock factor-binding protein 1 (HSBP1) regulates the levels of ATG13, RB1 inducible coiled-coil 1, and kinase unc-51 like autophagy activating kinase (ULK) 1, which are key components in regulating autophagy [105,106]. HSBP1 stabilizes the ULK complex and promotes autophagosome formation under starvation [105]. Nevertheless, HSBP1 depletion impaired EVA71 viral replication, even in ATG7-KO cells, where conventional autophagy was completely abolished [105]. This result suggests that HSBP1 possesses an autophagy-independent function in facilitating EVA71 infection [105]. These findings highlight the multifaceted roles of autophagy-related proteins in viral pathogenesis.

## 9. Conclusions

**Figure 2:**
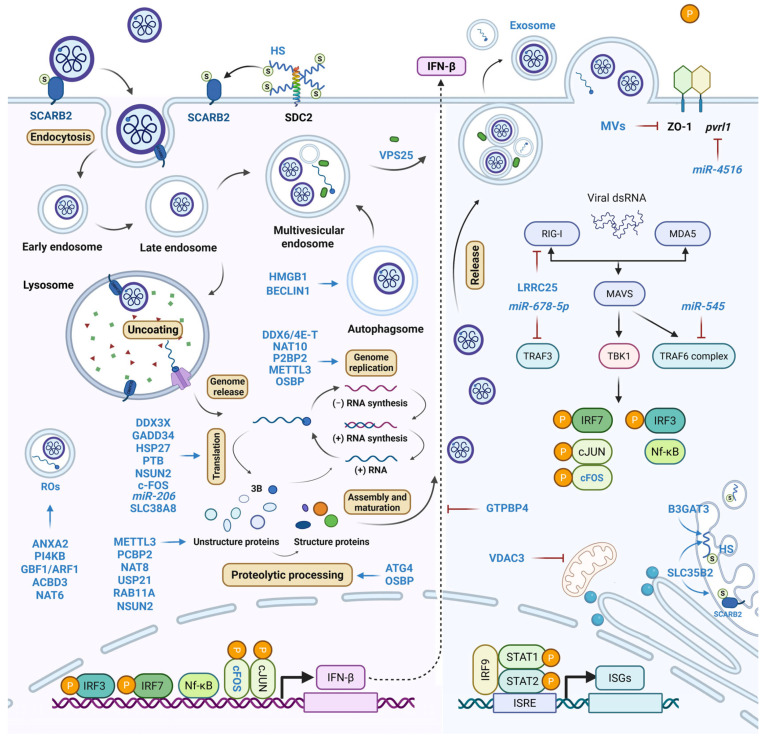
Pro-viral host factors during EVA71 infection. EVA71 composes a lifecycle of endocytosis, uncoating, genome release, genome replication, translation, assembly, and maturation, during which processes, host factors are hijacked to facilitate viral replication. The color-coded factors represent their predominant roles in specific stages of the viral life cycle. 3B indicates the non-structure protein of EVA71. 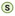 indicates sulfation and 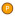 indicates phosphorylation.

Viruses utilize an array of host factors to regulate cellular processes upon infection and possess diverse functions. In this review, we summarized the newly identified pro-viral host factors and delineated their roles in the EVA71 lifecycle (Figure 2). The underlying mechanisms are systematically elucidated and compiled in Table 1. Nevertheless, complex mechanistic intricacies warrant further exploration. For instance, HSP27 is phosphorylated upon infection with herpes simplex virus 1 (HSV-1), and depletion of HSP27 reduces HSV-1 production [107]. Upon EVA71 infection, it also acts as a pro-viral factor that induces ITAF expression. However, during HBV infection, HSP27 acts as an antiviral factor by inducing the expression of type I interferons and downstream antiviral effectors (such as Interferon-stimulated gene 15, 2′-5′-oligoadenylate synthetase (OAS) 1, OAS3, protein kinase R, and eIF2α) [108]. This also applies to viruses belonging to the same genera. For example, both mTOR and p-mTOR levels are significantly reduced upon EV-D68 infection, whereas only the p-mTOR level is reduced after EVA71 infection [58,109]. Thus, certain factors may have opposing regulatory patterns within the same genus. miR-4516 was induced in coxsackievirus 16 (CVA16) infection and decreased in EVA71 infection, exerting different effects on downstream factors of PVRL1, claudin4, ZO-1, and E-cadherin and ultimately affecting airway epithelial integrity [86]. Therefore, it is important to investigate and elucidate the specific mechanisms of different viruses because of the complexity of cellular responses to viruses.

Although some molecular functions remain conserved within genera, such as PTB’s ITAF activity, which supports both Poliovirus and EVA71 replication [44,110], individual host factors often engage in pleiotropic signaling. DEAD-box helicase DDX6 exemplifies this duality by simultaneously promoting viral RNA synthesis and enhancing RIG-I-mediated IFN responses [35,111]. Autophagy pathways demonstrate context-dependent regulation and are typically hijacked by EVA71 to facilitate ROs formation and antiviral factor sequestration, which are frequently suppressed by other viruses to prevent viral protein degradation [77,112,113,114,115].

The intricate crosstalk that maintains cellular homeostasis is profoundly disrupted during viral infections. Central regulators, such as mTOR, integrate inputs from the eIF4E/eIF4G, ULK1, PI3K/AKT, and AMP-activated protein kinase pathways to coordinate cell growth, proliferation, and metabolism [61,116]. EVA71 exhibits multiple types of mTOR utilization. The SDLY107 strain induces autophagosome accumulation through p-mTOR suppression, enhancing viral translation and secretion [117]. Meanwhile, the O/BY/CHA/2010 strain requires mTOR-p70S6K-rpS6 signaling for aspartate-mediated viral translation, which is abrogated by rapamycin inhibition [58]. This discrepancy may reflect strain-specific adaptations or regulation. However, it is intriguing that p70S6K and rpS6 have been reported to cooperate with increased cap-dependent translation, a target usually compromised upon enterovirus infections, to compete with host mRNAs for translation factors and ribosomes [118,119]. In contrast, the Newcastle disease virus, an-ssRNA virus that harbors RNA capping and methylation abilities, activates mTOR to support cap-dependent translation for viral production [118].

## 10. Perspectives

Although translating fundamental discoveries of virus–host interactions into clinical applications remains a challenging process, mechanistic studies provide indispensable insights for the development of antiviral drugs and vaccines. The elucidation of viral receptor recognition can inform vaccine design strategies and neutralization antibody testing, as exemplified by the development of SARS-CoV-2 mRNA vaccines and the application of neuraminidase-receptor specificity in animal influenza A virus vaccines [120,121,122]. Furthermore, understanding immune evasion mechanisms supports vaccine optimization by enabling unfavorable antigens that compromise immunization to be avoided [123]. For instance, the conserved S2 subunit of SARS-CoV-2 antagonizes the IFN response by competitively binding to Signal transducer and activator of transcription 2 and blocking Interferon-stimulated gene factor 3 nuclear translocation [120]. Otherwise, therapeutic drugs can be screened from candidates that target virus–host interactions. Platforms such as ABPs, ChIRP-MS, organoid-based infection platforms, and CRISPR-Cas9 systems enable the systematic identification of a broad array of candidate drugs [51,97,124,125,126].

Concerning enteroviruses, which pose significant global health challenges due to their rapid mutation rates, frequent genetic recombination, and efficient transmission dynamics, vaccination remains one of the most effective strategies for preventing infectious diseases. However, currently licensed vaccines for EVA71 exclusively target the C4 serotype [3]. Protection against other HFMD-causing enteroviruses, particularly CVA6, CVA10, and CVA16, is required when they are recognized as the causal strains [127,128,129]. Consequently, the development of multivalent vaccines has become an essential strategy for achieving comprehensive protection against HFMD. In addition to conventional inactivated vaccines, virus-like particle-based platforms based on spodoptera frugiperda insect cells and yeast have been developed [130,131,132]. As the intricate interplay between recombinant proteins and host factors may lead to remodulated cellular responses, protein engineering approaches can be employed to optimize immunogenicity.

Additionally, there are no commercially approved therapeutics targeting EVA71. Current clinical management remains dependent on canonical broad-spectrum agents, including ribavirin, spiramycin, azithromycin, and IFNs [133,134]. Nevertheless, the side effects of antiviral drugs, antibiotic resistance, clinical efficacy, and mechanisms of action [135], as well as inadequate therapeutic effects in severe cases (particularly neurological complications), require further consideration when used clinically. To address these challenges, broad-spectrum antiviral strategies have been developed based on novel targets: (i) evolutionarily conserved viral elements or (ii) essential eukaryotic host factors [136]. Notably, host-directed antivirals have emerged as particularly promising candidates, offering broad-spectrum protection against evolving EVA71 variants while maintaining high genetic barriers to resistance. Despite the advantages, development of relevant antiviral drugs still requires extensive experimental and clinical data to ensure compliance with associated safety standards when strategies of functional modulation over ablation, tissue-selective delivery, and dosage window optimization could be considered. Totally, this review summarizes the current knowledge on EVA71 host dependencies, which could contribute to the rational development of targeted antivirals and next-generation vaccines.

## Figures and Tables

**Table 1:** Pro-viral host factors and mechanisms during EVA71 infection.

Step	Factor	Function	Refs.
Viral entry	SCARB2	Receptor	[21]
	SLC35B2	Tyrosine sulfation of receptors	[21]
	B3GAT3	HS backbone biosynthesis	[21]
Viral RNA	PI4KB	ROs formation	[26,27,28]
	GBF1/ARF1	PI4KB recruitment	[28]
	ACBD3	PI4KB recruitment	[28]
	NAT6	Stabilizing ACBD3	[29]
	ANXA2	PI4P induction and viral 3D recruitment	[30]
	DDX6/4E-T	Viral RNA binding	[35]
	NAT10	ac4C modification of viral 5′ UTR	[38]
	PCBP2	Stabilizing viral RNA-3D interaction	[39]
Viral protein	DDX3X	Facilitating ribosome entry	[40]
	GADD34	Promoting IRES activity	[41]
	HSP27	Activating IRES-mediated translation	[42]
	PTB	Activating IRES-mediated translation	[44]
	c-FOS	Activating MEK/ERK signaling	[48]
	NAT8	Lysine acetylation of 2B, 3AB, and 3C	[49]
	USP21	Deubiquitinating 2A^pro^	[50]
	ATG4	3C^pro^ protease-like activity	[51]
	RAB11A	Acting as scaffold of viral proteins	[52]
	SLC38A8	Activating mTOR/p70S6K1 signaling	[58,60]
	*miR-206*	Repressing G3BP2 expression	[62]
Viral RNA and protein	METTL3	Sumoylation and ubiquitination of 3D	[36]
	NSUN2	m5C modifications of EVA71 RNAs; stabilizing VP1	[45]
	OSBP	Promoting 3AB cleavage and plus-strand RNA synthesis	[65]
	LRRC25	Degradation of G3BP1 and RIG-I to inhibit stress granule formation and innate immunity	[76]
Immune evasion	GTPBP4	Negative regulator of IFNβ	[77]
	*miR-545*	Inhibiting IFN signaling via PENT and TRAF6	[78]
	*miR-628-5p*	Targeting TRAF3 to impair innate immunity	[79]
	VPS25	Promoting exosome biogenesis and secretion	[83]
Packaging and dissemination	*miR-4516*	Reducing *pvrl1* mRNA to disrupt CAMs	[86]
	BECLIN1	ATG protein recruitment	[96]
	HMGB1	Regulating autophagosome formation	[97]

## Data Availability

Not applicable.

## Abbreviations

The following abbreviations are used in this manuscript:

EVA71	Enterovirus A71
HFMD	Hand, foot, and mouth disease
UTR	Untranslated region
SLs	Stem loops
IRES	Internal ribosome entry site
ITAFs	IRES transfactors
HSP	Heat shock protein
SCARB2	Scavenger receptor class B member 2
SLC35B2	Solute carrier family 35 member B2
B3GAT3	Beta-1, 3-glucuronyltransferase 3
HS	Heparan sulfate
ROs	Replication organelles
PI4KB	Phosphatidylinositol-4-kinase IIIβ
PI4P	Phosphatidylinositol 4-phosphate
ARF1	ADP-ribosylation factor 1
GTPase	Guanosine triphosphatase
GBF1	Golgi Brefeldin A-resistant guanine nucleotide exchange factor 1
ACBD3	Acyl-coenzyme A-binding domain containing 3
NAT	N-terminal acetyltransferase
ANXA2	Annexin A2
RNP	Ribonucleoprotein
DDX	DEAD-box helicase
4E-T	eIF4E-transporter
METTL3	Methyltransferase-like 3
NAT10	N-acetyltransferase 10
PCBP2	Poly(rC)-binding protein 2
DDX3X	DEAD-box helicase 3 X-linked
GADD34	DNA damage-inducible protein 34
eIF2α	Eukaryotic translation initiation factor 2α
hnRNP A1	heterogeneous nuclear ribonucleoprotein A1
NSUN2	NOP2/Sun RNA methyltransferase family 2
USP	Ubiquitin-specific protease
ABPs	Activity-based probes
PTB	Polypyrimidine tract-binding protein 1
ER	Endoplasmic reticulum
ATG	Autophagy-related protein
RAB11A	RAS-associated protein 11A
TCA	Tricarboxylic acid cycle
PI3K	Phosphoinositide 3-kinase
AKT	Protein kinase B
SLC38A8	Solute carrier family 38 member 8
p70S6K1	Ribosomal protein S6 kinase beta-1
rpS6	Ribosomal protein S6
G3BP2	Stress granule assembly factor 2
OSBP	Oxysterol-binding protein
VDAC3	Voltage-dependent anion channel 3
ROS	Reactive oxygen species
RIG-I	Retinoic acid-inducible gene I
MAVS	Mitochondrial antiviral-signaling protein
IFN	Interferon
IRF	IFN response factor
G3BP1	Ras GTPase-activating protein-binding protein
LRRC25	Leucine-rich repeat-containing 25
YTHDF2	YTH N6-methyladenosine RNA binding protein F2
GTPBP4	GTP-binding protein 4
PTEN	Phosphatase and tensin homolog
TRAF	Tumor necrosis factor receptor-associated factor
EVs	Extracellular vesicles
MVs	Microvesicles
BBB	Blood–brain barrier
CNS	Central nervous system
ZO-1	zonula occludens-1
VPS	Vacuolar protein sorting
PVRL1	Poliovirus receptor-related 1
AJs	Adherens Junctions
BECLIN1	BCL-2 interacting protein 1
HMGB1	High mobility group box 1
HSBP1	Heat shock factor-binding protein 1
ULK	Kinase unc-51-like autophagy-activating kinase
HSV-1	Herpes simplex virus 1
OAS	2′-5′-oligoadenylate synthetase
CVA16	Coxsackievirus 16
